# Babesiosis-Induced Hemophagocytic Lymphohistiocytosis Following Spontaneous Splenic Rupture in a Florida Resident: A Case Report

**DOI:** 10.7759/cureus.83360

**Published:** 2025-05-02

**Authors:** Abeer Jacob, Samir A Nacer, Nikesh Shah

**Affiliations:** 1 Pathology, Trinity Medical Sciences University School of Medicine, Roswell, USA; 2 Pathology, University of South Florida Morsani College of Medicine, Tampa, USA; 3 Hematology and Oncology, Tampa General Hospital, Tampa, USA

**Keywords:** babesia micoti, blood pathology, hemophagocytic lymphohistiocytosis (hlh), tick-borne infections, zoonotic infectious disease

## Abstract

Babesiosis is a tick-borne protozoal infection caused by *Babesia* species, typically endemic to the northeastern United States. Cases in non-endemic regions, such as Florida, are exceptionally rare but can lead to severe complications if not promptly recognized. We report a case of hemophagocytic lymphohistiocytosis (HLH) secondary to babesiosis in a Florida resident who presented with spontaneous splenic rupture, a rare sequela of babesiosis. This patient, originally from New York, had a recent travel history to Maine, emphasizing the critical importance of a thorough epidemiological history in non-endemic areas. Diagnostic challenges were compounded by overlapping clinical features of HLH and sepsis. Key laboratory findings included anemia, thrombocytopenia, hyperferritinemia, and elevated inflammatory markers, with confirmatory diagnosis of babesiosis made via polymerase chain reaction (PCR) and peripheral blood smear. The patient responded well to atovaquone and azithromycin, with complete resolution of HLH symptoms and laboratory abnormalities. This case highlights the necessity for clinical vigilance, the integration of travel history into diagnostic evaluations, and the potential for fatal outcomes if babesiosis-induced HLH is not promptly diagnosed and treated. Increased awareness and training for clinicians in non-endemic regions, as well as public health measures, are essential to improving outcomes.

## Introduction

Hemophagocytic lymphohistiocytosis (HLH) is a rare, life-threatening hyperinflammatory syndrome characterized by dysregulated immune activation. This condition arises from impaired cytotoxic function of natural killer (NK) cells and cytotoxic T lymphocytes, leading to excessive macrophage and CD8+ T-cell activation [1]. An ensuing cytokine storm contributes to severe systemic inflammation and tissue injury, commonly manifesting as fever, cytopenias, splenomegaly, and multiorgan failure [1,2]. Mutations in genes that encode for perforins or those essential for lysosomal trafficking have been implicated in cytotoxic T-cell dysfunction [1]. Inciting causes of secondary HLH include infections (e.g. HIV, Epstein-Barr virus (EBV) and cytomegalovirus (CMV)), malignancies (typically hematologic such as lymphomas), and chronic immunosuppression [1].

Babesiosis, a tick-borne protozoal infection caused by *Babesia *species, is primarily transmitted by the *Ixodes scapularis* tick vector in endemic areas such as the northeastern and upper midwestern United States. *Babesia* infects erythrocytes through merozoite invasion, causing hemolysis and resulting in clinical manifestations ranging from asymptomatic infection to severe, life-threatening conditions. These can include retinal hemorrhages, congestive heart failure, gait ataxia, pancytopenia, acute liver failure, acute respiratory failure, and anemia [3]. Complications of babesiosis are more pronounced in individuals with asplenia, immunosuppression, or underlying health conditions [3,4].

*Babesia *has over 100 species, with only a few primarily associated with human infections. Key species include *B. divergens, B. microti, *and* B. duncani*. The epidemiology of *Babesia* is expanding, particularly in 10 high-risk states, including New York. Transmission of *Babesia *through vertical transmission and blood transfusions has been documented [5-10]. A significant clinical barrier to care is the co-infection of *Babesia* with other tick-borne illnesses, such as Lyme disease. Since treatment regimens for these conditions differ, co-testing is essential when parasitosis is suspected [11].

Although HLH and babesiosis are distinct entities, babesiosis serves as an infectious and potent trigger for secondary HLH, particularly in individuals with compromised immune regulation [3]. The diagnostic challenges posed by overlapping clinical and laboratory features often lead to delays in recognition and treatment [3]. In this report, we describe a unique case of babesiosis-induced HLH in a Florida resident and New York native who presented with nonspecific symptoms following travel to an endemic region. Our aim is to add to the growing literature on diverse presentations of babesiosis infection, underscoring the importance of integrating epidemiological history with clinical findings for timely diagnosis and management.

## Case presentation

**Figure 1:**
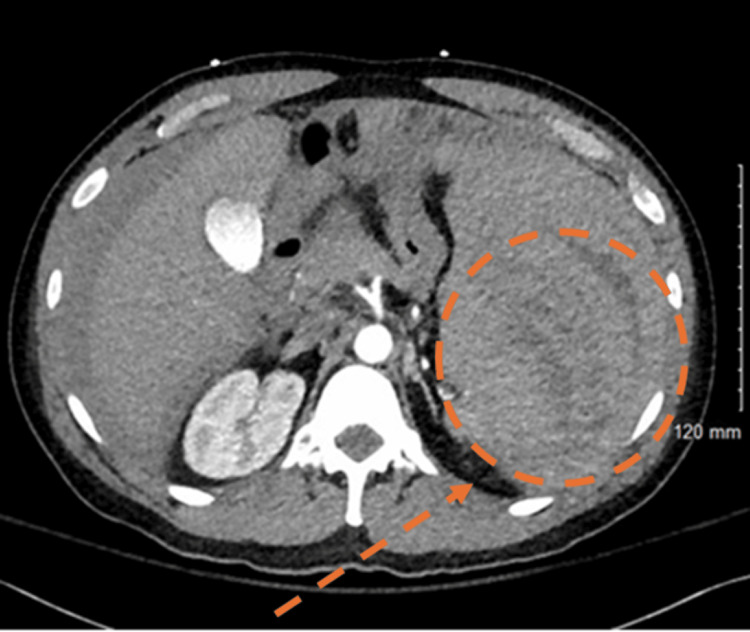
Transverse CT scan of the abdomen Highlights a splenic rupture, with evidence of disrupted splenic parenchyma and associated changes suggestive of intra-abdominal hemorrhage. The affected area is circled for clarity.

**Figure 2:**
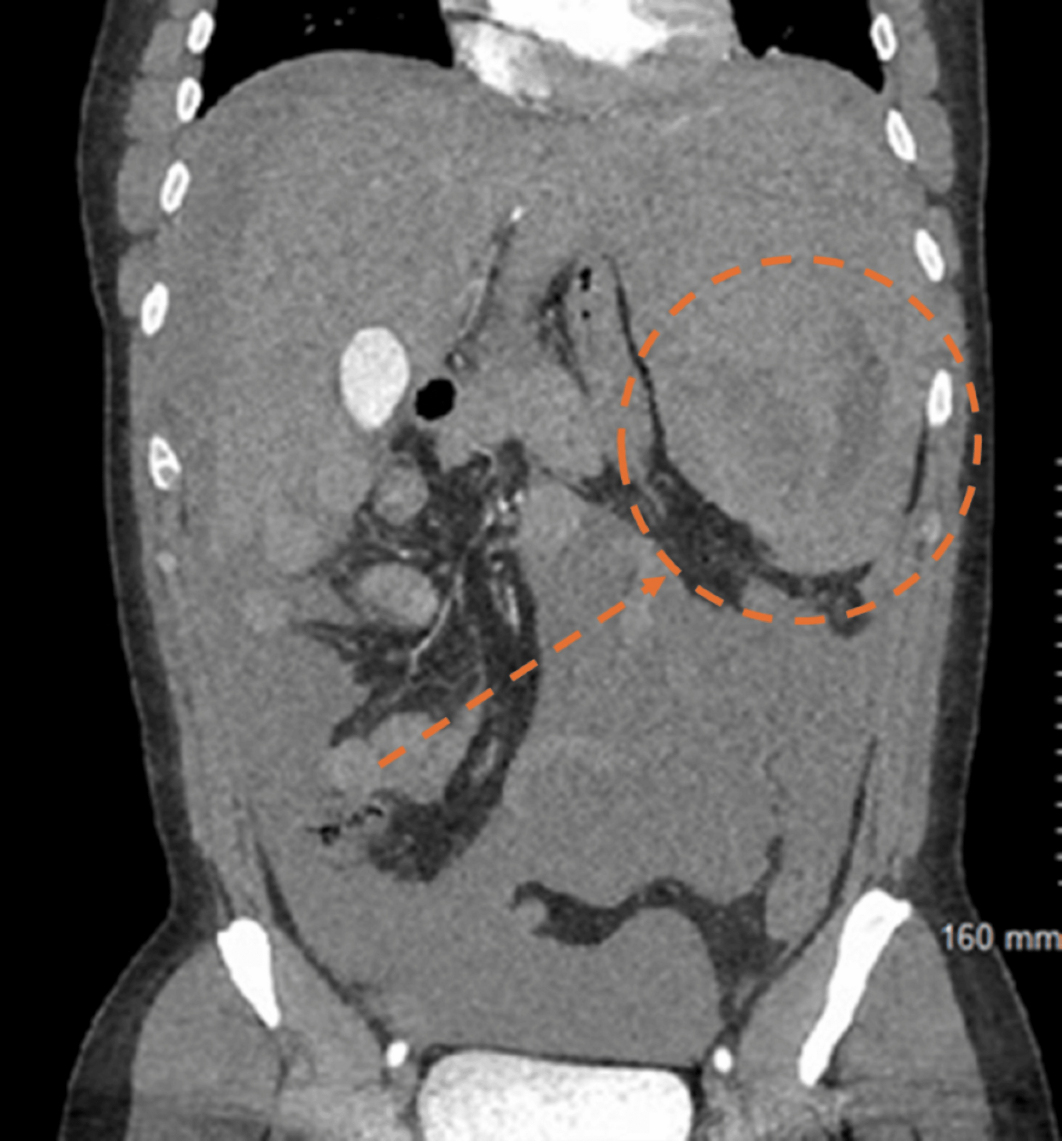
Coronal CT scan of the abdomen and pelvis. Demonstrates a splenic rupture with associated findings, including evidence of intra-abdominal hemorrhage and splenic parenchymal disruption.

A 44-year-old previously healthy Caucasian male originally from New York, currently residing in Florida, presented to his local emergency department (ED) with complaints of diffuse abdominal pain and dizziness. He denied fever, vomiting, diarrhea, urinary symptoms, or syncope. Vital signs showed no evidence of hypotension or shortness of breath. Following imaging investigations, he was found to have a large amount of hemoperitoneum and a large perisplenic hematoma (Figures 1, 2). Hemoglobin of 7.3 g/dL was discovered on complete blood count. He received two units of packed red blood cells before being transferred to another facility for emergent splenectomy. Following splenectomy, during the same admission, he was found to have mild leukocytosis, anemia, and thrombocytopenia, which was treated with multiple blood transfusions. He was discharged a few days later with prescribed prophylactic penicillin.

**Figure 3:**
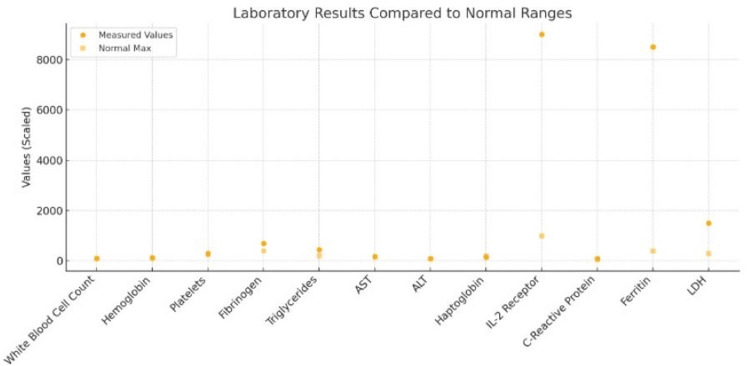
Laboratory results Laboratory values at hospital re-admission following splenectomy reveal pancytopenia, indicated by reductions in white blood cell count, hemoglobin, and platelets. In contrast, inflammatory markers such as IL-2 receptor, ferritin, and lactate dehydrogenase (LDH) are markedly elevated, exceeding normal maximums by several fold. These findings are consistent with a systemic inflammatory response and potential immune dysregulation post-splenectomy. ALT: alanine aminotransferase; AST: aspartate aminotransferase

Twelve days later, he received his post-splenectomy vaccinations. The following day he began to have fever, malaise, chills, shortness of breath, and mild scleral icterus lasting a duration of six days, which prompted him to return to the ED. Physical examination revealed normal findings aside from constitutional symptoms. Initial laboratory studies were notable for pancytopenia, with a hemoglobin of 9.1 g/dL, platelet count of 34,000/μL, and white blood cell count of 3,200/μL, serum interleukin-6 <5.00 pg/mL, along with elevated inflammatory markers and liver function tests (total bilirubin 2.3 mg/dL, direct bilirubin 1.5 mg/dL, albumin 3.8 g/dL, and alkaline phosphatase 210 U/L) (Figure 3). Over the subsequent hospital course, the white blood cell count rose to 14,500/μL in the context of systemic inflammation. Prothrombin time (PT), partial thromboplastin time (PTT), international normalized ratio (INR) and electrolytes were unremarkable. Computed Tomography scan of the thorax, abdomen, and pelvis (CT TAP) and CT chest revealed no acute findings. Empiric treatment with vancomycin and ceftriaxone were initiated for suspected sepsis. Due to high suspicion for HLH based on the findings in Table 1, dexamethasone was administered prior to transferring to a tertiary care center where Malignant Hematology was consulted for further evaluation and management for suspected HLH.

**Table 1:** H-score Summary of the patient's H-score calculation of 233, which indicates a 98-99% probability of hemophagocytic lymphohistiocytosis (HLH), exceeding the diagnostic cutoff of ≥169. Each parameter contributing to the score, along with its measured value and diagnostic threshold, is detailed to highlight the clinical findings supporting the diagnosis. AST: aspartate aminotransferase

Parameter	H-score Points	Measured Value	Diagnostic Threshold
Known underlying immunosuppression	18	Yes	Known immunosuppression
Splenomegaly	23	Present	Present
Two lineages of cytopenia	24	Hemoglobin 9.1 g/dL	Hemoglobin ≤ 9.2 g/dL
-		Platelets 34,000/µL	Platelets ≤ 110,000/µL
Ferritin >6000 µg/L	50	8,413 µg/L	>6000 µg/L
Triglycerides >4 mmol/L	64	21.9 mmol/L	>4 mmol/L
AST ≥ 30 U/L	19	122 U/L	≥ 30 U/L
Hemophagocytosis on bone marrow aspirate	35	Present	Present
Total H-score	233	-	Diagnostic cutoff: ≥ 169

**Figure 4:**
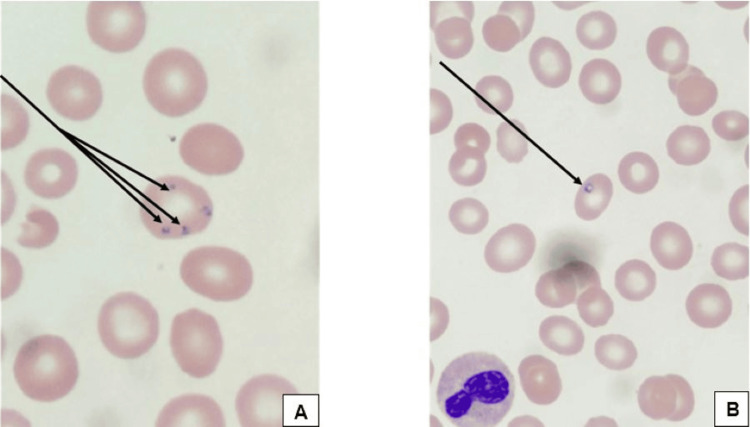
Peripheral blood smear On Wright Giemsa stain, demonstrating (A-B) intracellular ring forms consistent with babesiosis (Arrows).

On arrival at the tertiary care center, a broad hematologic and infectious work-up was initiated for malignancy and microbial causes. A peripheral blood smear revealed intracellular ring forms consistent with babesiosis (Figure 4). Corresponding flow cytometric analysis revealed no immunophenotypic abnormalities of the lymphoid cells or myeloid cells and no evidence of malignancy; no blastosis was detected. The patient did not undergo red blood cell exchange transfusion due to minimal evidence of parasitemia burden observed on blood smear. *Plasmodium falciparum malaria, Toxoplasma gondii*, and *Candida* *auris* serologies were negative. Confirmatory testing with serum polymerase chain reaction (PCR) proved positive for *Babesia*. Following further inquiry, the patient revealed he had taken a recent hiking trip in Maine.

**Figure 5:**
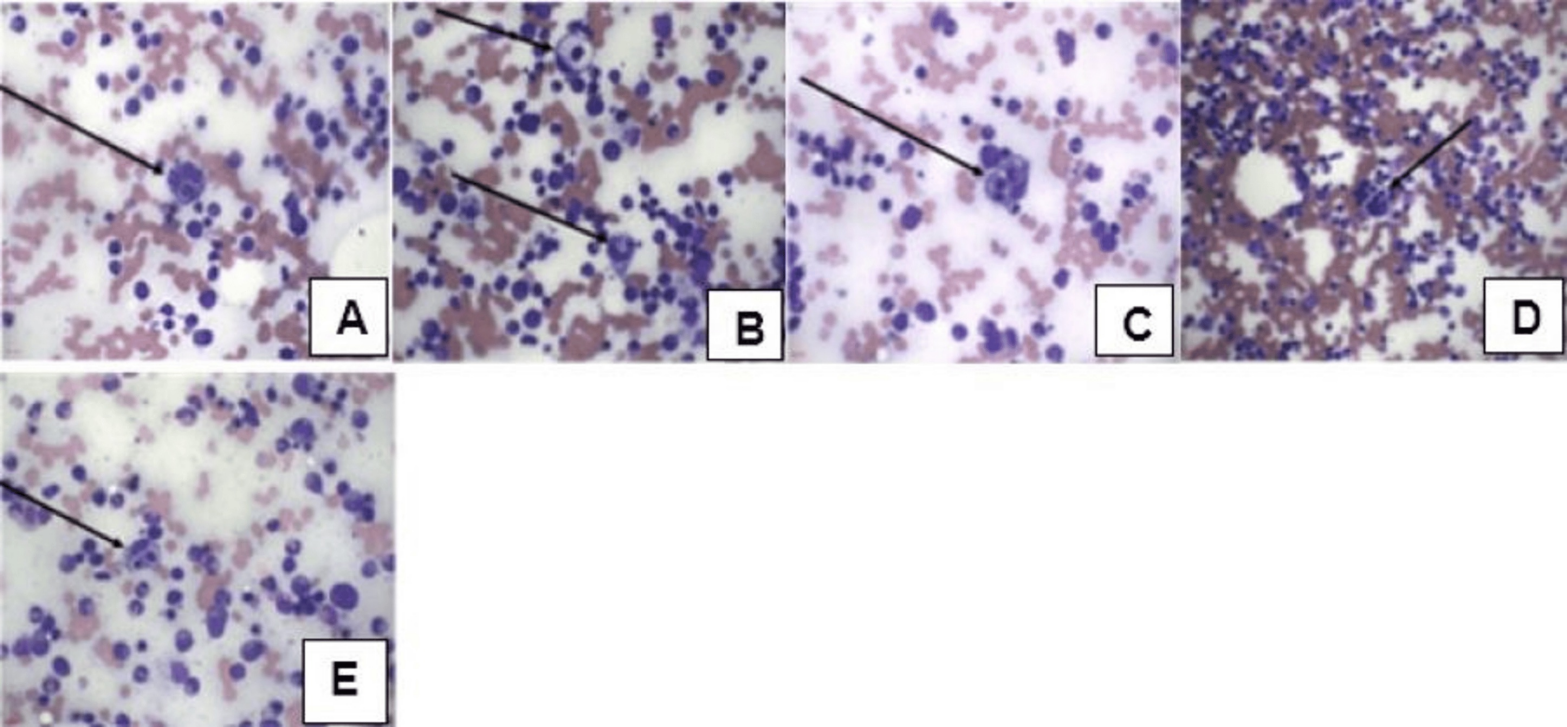
Bone marrow biopsy Hemophagocytic Lymphohistiocytosis (HLH), including evidence of (A-E) hemophagocytosis with macrophages (Arrows) engulfing red blood cells, lymphocytes, and neutrophils.

Further hematologic and infectious work-up was negative for human immunodeficiency viruses, methicillin-resistant *Staphylococcus aureus, Legionella pneumophila, Histoplasma capsulatum*, and EBV. Furthermore, panels for hepatitis A, B, and C antibodies were nonreactive, Cryptococcal antigen was negative, and *Mycoplasma pneumoniae* (IgM) was undetectable. A bone marrow biopsy showed no evidence of malignancy but revealed hypercellular marrow with hemophagocytosis consistent with HLH (Figure 5). H-score was calculated to be 233 points which equates to a 98-99% probability of hemophagocytic syndrome (Table 1); 18 points for known underlying immunosuppression, 23 points for splenomegaly, 24 points for two lineages of cytopenia as evidenced by patient’s hemoglobin of 9.1 g/dL and platelet count of 34,000/μL (hemoglobin ≤ 9.2 g/dL and platelets ≤ 110,000/μL, 50 points for ferritin >6000μg/l (8413μg/l), 64 points for triglycerides >4 mmol/L (21.9 mmol/L), 19 points for aspartate aminotransferase (AST) ≥ 30 U/L (122 U/L), and 35 points for hemophagocytosis features on bone marrow aspirate further supporting HLH [12].

Final diagnosis was concluded to be HLH secondary to babesiosis. A 10-day course of atovaquone and azithromycin was initiated, and inflammatory markers were followed for complete resolution. The patient's presenting symptoms, leukocytosis, anemia, thrombocytopenia, and elevated inflammatory markers resolved after four days of treatment (Table 2).

**Table 2:** Inflammatory and hematologic markers Trends in inflammatory and hematologic markers during treatment for secondary hemophagocytic lymphohistiocytosis (HLH) with antibiotic therapy. Values indicate elevated levels (H) for markers such as lactate dehydrogenase (LDH), C-reactive protein, ferritin, and triglycerides across specific dates.

Date	LDH (125 - 220 U/L)	C-Reactive Protein (0 - 0.5 mg/dl)	Ferritin (21.8 - 274.7 ng/mL)	Triglycerides (<150.0 mg/dL)
8/8/2024	1075 (H)	-	4765.5 (H)	-
8/9/2024	1003 (H)	6.35 (H)	-	394 (H)
8/10/2024	901 (H)	4.21 (H)	2256.3 (H)	-
8/11/2024	902 (H)	2.83 (H)	2432.0 (H)	-
8/12/2024	729 (H)	1.9 (H)	2024.5 (H)	-

Post-splenectomy precautions and vaccination against *Streptococcus pneumoniae, Haemophilus influenzae, Neisseria meningitidis* were completed [13]. The patient demonstrates significant clinical improvement at the most recent follow-up, with complete resolution of symptoms and marked improvement in blood counts and inflammatory markers.

## Discussion

*Babesia*, while a known risk factor for HLH, is an uncommon cause of this severe and potentially life-threatening condition. This case represents a rare instance of HLH triggered by *Babesia*, consistent with a small but growing body of literature documenting babesiosis as a potential etiology of HLH [4]. The excessive inflammation induced by *Babesia* leads to a cytokine storm, with elevated pro-inflammatory markers like interleukin-1 (IL-1), interleukin-6 (IL-6), and tumor necrosis factor-alpha (TNF-α), driving systemic immune dysregulation [1]. This immune response can trigger HLH, where hyperactivated macrophages and cytotoxic T cells damage healthy tissues, causing severe complications such as pancytopenia, organ failure, and coagulopathy [3]. Importantly, the uniqueness of this case also lies in the patient’s initial presentation with spontaneous splenic rupture, which led to the emergent splenectomy. This rare and dramatic presentation was later understood to be a consequence of splenomegaly, likely related to early *Babesia *infection, making it a critical clinical clue in the diagnostic trajectory.

We present a unique case of HLH triggered by *Babesia*, characterized by the constellation of fever, malaise, chills, shortness of breath, mild scleral icterus. Initial laboratory findings at re-admission demonstrated pancytopenia, including anemia, thrombocytopenia, and leukopenia. This was followed by the development of leukocytosis, likely in response to systemic inflammation and suspected sepsis. Additional findings included hyperferritinemia, hypertriglyceridemia, alongside imaging that revealed pathologic splenic rupture. The case is further distinguished by its unusual initial presentation with spontaneous splenic rupture, a rare but serious manifestation that may reflect early splenomegaly associated with underlying *Babesia *infection.

Findings on peripheral blood smear showed merozoites in clusters inside RBCs, and evidence of histiocytes with vacuoles engulfing red blood cells, lymphocytes, and neutrophils on bone marrow biopsy consistent with HLH. A positive Babesia PCR, the gold standard for diagnosis, confirmed the infection, as it is more sensitive than microscopy or peripheral blood smear [14]. Further investigation revealed that this patient, presenting in Florida and originally from New York, had a recent travel history to Maine.

HLH secondary to babesiosis can be easily overlooked due to its nonspecific symptoms and clinical overlap with other conditions, such as sepsis or hematologic malignancies. This emphasizes the need for heightened clinical vigilance, thorough history-taking - including travel and exposure histories - and a multidisciplinary approach to ensure timely and accurate diagnosis. Although the non-endemic risk of *Babesia* exposure remains low, Florida's diverse and transient population necessitates consideration of *Babesia* as part of the differential diagnosis [15-17]. Patient interviews should include questions about recent travel to endemic areas, exposure to ticks, or activities that may increase risk, such as hiking or outdoor work in regions with high tick populations. Migration-related transmission should also be considered, highlighting the importance of inquiring about recent blood transfusions or coexisting tick-borne illnesses. These proactive steps are crucial for identifying potential cases in states with a dynamic and growing population profile [9].

A further complication in the diagnosis of *Babesia*-induced HLH is the potential for pre-treatment therapies to obscure key diagnostic markers. In this case, had the patient received empiric treatment with antibiotics such as azithromycin or doxycycline prior to presentation, it could have interfered with the clinical diagnosis by suppressing inflammatory responses. When referencing inflammatory values and surveying to find clues of babesiosis, it is important to note that corticosteroids are commonly used not for babesiosis itself, but in cases of suspected HLH, a severe hyperinflammatory syndrome. In this case, corticosteroids were initiated empirically due to concern for HLH, which can present with overwhelming immune activation and systemic inflammation. Their use aimed to temper the cytokine-driven immune dysregulation, rather than to treat babesiosis or presumed sepsis. This distinction is important, as corticosteroids are not indicated for babesiosis and are generally reserved in sepsis for cases involving refractory shock requiring vasopressors [18]. However, a low IL-6 does not remove HLH from the differential, and this is highlighted by the notable H-Score and the multiple components that are incorporated to calculate it [4,18-20].

Mitigating potential long-term complications of both babesiosis and HLH, such as recurrent hemophagocytic episodes, persistent cytopenias, organ dysfunction (e.g., liver or renal impairment), and increased risk of opportunistic infections requires monitoring for systemic symptoms like fever, fatigue, or myalgias and frequent laboratory tests to monitor ferritin, triglycerides, anemia, and leukocytosis [3,21-23]. Blood smears may be reviewed to monitor signs of hemophagocytosis or recurrent parasitemia. Regular follow-up visits with hematology and infectious disease specialists help to track HLH progression or remission.

## Conclusions

This case highlights the rarity of *Babesia *as a cause of HLH and the need for a broad differential diagnosis in patients with relevant travel history. It also draws attention to spontaneous splenic rupture as an exceptionally rare presentation of babesiosis - warranting thorough evaluation for infectious causes when trauma is absent. Earlier recognition at the time of rupture might have expedited diagnosis and treatment. This report adds to the growing literature on atypical babesiosis presentations and emphasizes the importance of integrating epidemiologic context with clinical findings to guide timely diagnosis and management.
